# Homeostasis in the Gut Microbiota in Chronic Kidney Disease

**DOI:** 10.3390/toxins14100648

**Published:** 2022-09-20

**Authors:** Shruti Bhargava, Erik Merckelbach, Heidi Noels, Ashima Vohra, Joachim Jankowski

**Affiliations:** 1 Institute of Molecular Cardiovascular Research, Medical Faculty, RWTH Aachen University, 52062 Aachen, Germany; 2 Institute of Home Economics, Delhi University, Delhi 110021, India; 3 Experimental Vascular Pathology, Cardiovascular Research Institute Maastricht (CARIM), University of Maastricht, 6211 Maastricht, The Netherlands

**Keywords:** gut-derived uremic metabolites, alterations of the gut microbiome, gut permeability, pathogenic bacteria in CKD

## Abstract

The gut microbiota consists of trillions of microorganisms, fulfilling important roles in metabolism, nutritional intake, physiology and maturation of the immune system, but also aiding and abetting the progression of chronic kidney disease (CKD). The human gut microbiome consists of bacterial species from five major bacterial phyla, namely *Firmicutes*, *Bacteroidetes*, *Actinobacteria*, *Proteobacteria*, and *Verrucomicrobia*. Alterations in the members of these phyla alter the total gut microbiota, with a decline in the number of symbiotic flora and an increase in the pathogenic bacteria, causing or aggravating CKD. In addition, CKD-associated alteration of this intestinal microbiome results in metabolic changes and the accumulation of amines, indoles and phenols, among other uremic metabolites, which have a feedforward adverse effect on CKD patients, inhibiting renal functions and increasing comorbidities such as atherosclerosis and cardiovascular diseases (CVD). A classification of uremic toxins according to the degree of known toxicity based on the experimental evidence of their toxicity (number of systems affected) and overall experimental and clinical evidence was selected to identify the representative uremic toxins from small water-soluble compounds, protein-bound compounds and middle molecules and their relation to the gut microbiota was summarized. Gut-derived uremic metabolites accumulating in CKD patients further exhibit cell-damaging properties, damage the intestinal epithelial cell wall, increase gut permeability and lead to the translocation of bacteria and endotoxins from the gut into the circulatory system. Elevated levels of endotoxins lead to endotoxemia and inflammation, further accelerating CKD progression. In recent years, the role of the gut microbiome in CKD pathophysiology has emerged as an important aspect of corrective treatment; however, the mechanisms by which the gut microbiota contributes to CKD progression are still not completely understood. Therefore, this review summarizes the current state of research regarding CKD and the gut microbiota, alterations in the microbiome, uremic toxin production, and gut epithelial barrier degradation.

## 1. Introduction

### 1.1. Mutualism between the Gut Microbiota in Healthy Individuals

The gut microbiome has recently emerged as a significant contributing factor to the overall health of an individual, contributing to both the prevention and progression of various diseases such as chronic kidney disease (CKD) and its associated complications including cardiovascular diseases (CVD) [1,2].

The human gut microbiome consists of 10–100 trillion microorganisms with more than 400 bacterial species from five major bacterial phyla, namely *Firmicutes*, *Bacteroidetes*, *Actinobacteria*, *Proteobacteria*, and *Verrucomicrobia* [3,4,5]. *Firmicutes* (*Ruminococcus*, *Clostridium*, *Lactobacillus*, *Eubacterium*, *Faecalibacterium*, and *Roseburia*) and *Bacteroidetes* (*Bacteroides*, *Prevotella*, and *Xylanibacter*) are the most predominant in healthy individuals [6].

The *Firmicutes* include Gram-positive bacteria with a low guanine/cytosine (G/C) content in their DNA. Most of the species have a rod-shaped morphology (straight or slightly curved) and cell walls containing muramic acid. Many members of this phylum break down complex carbohydrates in the gut that cannot be digested by endogenous enzymes [7]. *Lactobacillus*, a probiotic bacterium, found in fermented dairy products, belongs to this phylum and leads to short-chain fatty acid (SCFA) production, such as acetate, lactate, and antimicrobial molecules that prevent the colonization of the gut by pathogens [8]. Besides *Lactobacillus*, other mutualistic bacteria of the *Firmicutes* phylum, such as *Faecalibacterium*, *Eubacterium*, *Roseburia*, and *Anaerostipes* ferment carbohydrates to produce butyrate which acts as an energy source for the host and has anti-carcinogenic, and anti-inflammatory effects [7].

*Bacteroidetes* are anaerobic Gram-negative bacteria that colonize the entire gastrointestinal tract. One of the most prevalent genus of this phylum, *Bacteroides* species are anaerobic, bile-resistant, non-spore-forming, Gram-negative rods [9]. They ferment complex carbohydrates and produce volatile fatty acids which are a source of energy for the host [9]. Another significant bacterial genus in the gut belonging to the phylum *Bacteroidetes* is, *Prevotella*. Prevalence of *Prevotella* is higher in individuals consuming a plant-based diet as compared to those consuming largely animal diets [10]. This high-fiber diet acts as a prebiotic and stimulates the growth of mutualistic bacteria. The gut microbiomes of Americans and Europeans consuming a Western diet tend to be dominated by *Bacteroides* and Clostridiales, while rural populations with a high-fiber, low-protein diet tend to be dominated by *Prevotella* [11]. However, studies have also shown that *Prevotella* colonization results in metabolic changes in the microbiota, leading to reduced IL 18 production and consequent increase in susceptibility to mucosal inflammation, and potential systemic autoimmunity [12].

The ratio of *Firmicutes/Bacteroidetes* (F/B) plays an important role in maintaining intestinal homeostasis [13] and a deviation of F/B ratio is regarded as dysbiosis which leads to pathological conditions [14]. While some members of both *Firmicutes* and *Bacteroidetes* are probiotics, an overall increase in *Firmicutes* coincides with obesity and an increase in *Bacteroidetes* coincides with inflammatory bowel disease [14]. The F/B ratio is of importance as some members of one phyla help maintain normal numbers of potential pathogenic bacteria from the other phyla [14]. While the ideal F/B ratio is 1, it is known to increase with age in both males and females [15]. The F/B ratio is highly significant in CKD patients and related complications such as hypertension and obesity [16,17,18]. There is a decrease in microbial diversity, in addition to an increase in F/B ratio under CKD and hypertensive conditions [16,18]. In addition to the increase in F/B ratio, the abundance of acetate- and butyrate-producing bacteria was reduced [16]. Under CKD conditions, a high-fiber diet increased microbial biodiversity and the abundance of *Bacteroidetes* leading to a lower F/B ratio. This was accompanied by lower concentrations of indoxyl sulfate and p-cresol sulfate. This leads to improved kidney function highlighting the importance of a balanced F/B ratio in maintaining kidney health [18]. High-fiber diet intake leads to enrichment of SCFA-producing bacteria, balancing the F/B ratio by increasing the *Bacteroidetes* [19].

*Actinobacteria* are a phylum of mostly Gram-positive bacteria which comprise a small percentage of the gut microbiota but are essential for gut homeostasis. An *Actinobacteria*, *Bifidobacteria* is widely used as a probiotic, validating the importance of these phyla in the maintenance of gut homeostasis [20].

*Proteobacteria* constitute a small percentage of the gut microbiota in healthy individuals and are characteristically facultative anaerobes. *Proteobacteria*, including common bacteria such as *Escherichia coli* and *Salmonella*, are increased in dysbiosis conditions. An increase in *Proteobacteria* often coincide with a compromised gut microbiota and inflammation [21].

*Verrucomicrobia* is in comparison a small phylum of Gram-negative bacteria with the mucus-degrading bacterium *Akkermansia muciniphila* as the only member of this phylum identified in the human gut microbiota [22]. An increase in *Akkermansia muciniphila* is associated with a healthy gut due to its ability to improve gut barrier function and has anti-inflammatory properties [22]. The potential members and the functions of the five major bacterial phyla of the human gut are summarized in Table 1. 

Under non-pathogenic conditions these microorganisms have a symbiotic relationship with their host, contributing to metabolism, nutrition and physiology, leading to various health benefits; a symbiotic gut microbiota prevents pathogenic bacterial colonization in the host by competing for nutrients, triggering and enhancing host immune responses and even by directly killing the pathogenic microbes by mechanisms such as phagocytosis [3]. The gut microbiota produces vitamin B12, riboflavin, biotin, nicotinic acid, thiamine and vitamin K among other metabolites essential for survival. The SCFAs produced by gut bacteria are a source of energy for the host, improving glucose homeostasis, maintaining gut integrity and function and are anti-inflammatory and anti-carcinogenic. The gut–colonizing bacteria also benefit the host by stimulating both humoral and cell-mediated immunity [23]. The gut microbiota furthermore modulates brain development, behavior and neural health [24]. This interaction is facilitated through the enteric nervous system and by defining the host immune cell’s function [24]. Alterations in the gut microbiota alter the hormonal secretions from intestinal epithelial cells which signal the enteric nervous system contributing to complications such as eating disorders, gastroparesis and irritable bowel syndrome [24].

The gut microbiota modifies the fermentation of substrates such as dietary fibers, production of SCFAs and gases (CO_2_, CH_4_, and H_2_) [25], and activation of intestinal gluconeogenesis in the gut [26]. SCFAs prevent obesity via ’G protein-coupled receptor’ GPR41 receptor by promoting the expression and activity of anorectic hormones such as glucagon-like peptide-1 and peptide YY, which are produced by colonic L-cells [27]. These hormones lead to perceived satiety and reduce food intake by acting on the hypothalamus [28]. SCFAs such as butyrate, propionate and acetate interact with metabolite-sensing ‘G protein-coupled receptors’ GPR41, GPR43 and GPR109A present in the gut epithelium and on immune cells contributing to host gut homeostasis [29] and maintain the intestinal epithelial barrier integrity [30].

### 1.2. Gut Microbiota Dysbiosis in Chronic Kidney Disease

Indigenous microbiota can change non-pathological microenvironments within the organism to disease-prone conditions by changing the balance between symbiotic microbes by pathogenic changes in the abundance of gut microflora belonging to the 5 major phyla [1]. Disease conditions such as a low glomerular filtration rate in CKD, in turn, lead to alterations of gut microflora by means of metabolite accumulation, consequently altering the intestinal epithelial barrier to mediate and promote a bias in gut microflora in an exponential feedforward loop [31]. Gut microbiota alterations in CKD conditions promote an increase in intestinal permeability facilitating an increment of endotoxins such as lipopolysaccharides (LPS) in blood [1], disrupting the blood homeostasis leading to atherosclerosis and an increase in mortality of CKD patients [32].

The fermentation of non-digestible substrates has certain health benefits, e.g., energy provision and contributing to glucose homeostasis [1]. However, protein substrates lead to the release of amines, indoles and phenols, the accumulation of which has adverse effects on CKD patients [1]. An increase in uremic metabolites due to gut-microflora leads to an increase in inflammation, oxidative stress and deimmunization [1,26,32]. In CKD patients, activation of the NF-ĸB pathway due to elevated levels of microbial metabolites such as p-cresol, trimethylamine and indole propionic acid leads to systemic inflammation [33,34] which translates to a high risk of atherosclerosis. The accumulation of uremic metabolites leads to reactive oxygen species (ROS) formation in the kidney epithelial cells by inflammasome-mediated IL-1β production [35].

Gut microbiota-mediated fermentation of substrates such as dietary phosphatidylcholine and carnitine leads to the production of trimethylamine in the gut [26]. Indole propionic acid, which is correlated with dietary fibers intake, is also produced by the gut microbiota [26]. While oxidized trimethylamine (trimethylamine N-oxide (TMAO)) disturbs the blood homeostasis in CKD patients by causing platelet hyperactivity and lipid disorders [32] by promoting metabolic bacteremia and endotoxemia and reducing the expression of angiopoietin-like protein 4 which inhibits lipoprotein lipase activity and stimulates white adipose tissue lipolysis [30], indole propionic acid is associated with a reduced risk of type 2 diabetes demonstrating the importance of the gut microbiota in determining the risk of chronic disorders [36].

It is essential to know the homeostasis of the intestinal microbiota in CKD. Therefore, this review summarizes the current state of research regarding CKD and the gut microbiota, alterations in the microbiome, uremic toxin production, and gut epithelial barrier degradation. A literature search was done for the gut microbiota in healthy individuals and alterations in the gut microbiota in CKD to form the basis of this review. Additionally, a list of gut-derived uremic toxins which have the highest global toxicity score in terms of several biological systems affected and overall experimental and clinical evidence was found in a recent study by Vanholder et al. [37]. A second literature search was carried out for each of the uremic metabolites from this list in terms of the microbes which influence these metabolites and their role in CKD and the impact on gut-derived uremic metabolites on gut health in terms of deimmunization and damage to the intestinal epithelial barrier.

## 2. Alterations in the Intestinal Microflora in Chronic Kidney Disease

Dysbiosis of intestinal flora in patients with CKD is associated with a decline in the number of symbiotic flora and an increase in certain indigenous microbes leading to pathogenic conditions, which may cause or aggravate CKD due to disorders of the metabolic, endocrine or immune system [38]. This alters the composition, diversity and richness of the microflora: phyla *Proteobacteria*, *Actinobacteria* and *Firmicutes* show overgrowth in the duodenum and jejunum [38].

The gut microbe *Akkermansia* from the phyla *Verrucomicrobia* plays a pivotal role in increasing gut-barrier function and thickness of the mucus [4] and helps in the detoxification of hydrogen sulfide [39]. It supports the growth of bacteria-producing SCFAs such as butyrate, by providing them with carbon, nitrogen and energy produced as a result of mucus degradation [40]. Studies on fecal microbial communities in CKD patients have revealed that the abundance of probiotic bacteria *Akkermansia* was decreased as compared to healthy controls [40].

The gut bacteria from phyla *Proteobacteria* are responsible for causing an inflammatory response, alteration of gut mucosal permeability and increasing the cell ratio of intestinal T helper 17 cells to T regulatory cells and promoting the LPS translocation. The gut microbiota of CKD patients showed an increase in members of the phylum *Bacteroidetes* and *Proteobacteria* and a decrease in *Lactobacillus*, which belongs to the phyla *Firmicutes*, compared to healthy individuals [41]. Lower abundance of *Lactobacillus* is associated with the development of hypertension and linked to adverse outcomes in patients with CKD [42].

Patients with CKD show enhanced numbers of phylum *Proteobacteria*, genus *Escherichia*, *Shigella*, *Desulfovibrio*, and phylum *Firmicutes*, genus *Streptococcus*, while a lower abundance of the phylum *Firmicutes*, genus *Roseburia*, *Faecalibacterium*, and *Prevotella* [43]. A less prominent bacteria, *Pyramidobacter* from the phylum *Synergistetes*, is also reduced [43]. Lower numbers of these bacteria lead to less production of butyrate a compound known to protect kidneys [44]. Butyrate inhibits the histone deacetylases, thereby reducing fibrosis and attenuating acute kidney injury-mediated damage [45]. It furthermore exhibits anti-inflammatory properties as an agonist for ´G protein-coupled receptors´, which is involved in inflammation regulation [22].

A comparison between the CKD group and the healthy controls showed that *Ruminococcus* and *Roseburia* from the phyla *Firmicutes* have the best diagnostic performance within the thirty-one species identified with a differentiated prevalence [46]. *Ruminococcus* promotes inflammatory bowel syndrome, promoting CKD-associated complications by producing inflammatory polysaccharides such as glucorhamnan which induce inflammatory cytokine tumor necrosis factor alpha secretion by dendritic cells [47]. *Roseburia* is reduced in inflammatory and metabolic diseases and produces butyrate, which has anti-inflammatory and immunoregulatory functions [48] leading to damaged local gastrointestinal tract function and thereby aggravating inflammation in CKD patients [49].

Members of the phylum *Verrucomicrobia* were reduced and *Actinobacteria* were increased in CKD than in the healthy control groups [4]. Minor differences were observed in the numbers of several other bacterial genera in CKD patients, including *Parasutterella* from phyla *Proteobacteria*, *Paraprevotella* from the phyla *Bacteroidetes*, *Clostridium IV* from the phyla *Firmicutes*, and *Alloprevotella* from the phyla *Bacteroidetes* [39,50].

In CKD patients, *Bifidobacterium* from the phyla *Actinobacteria* was depleted and supplementing *Bifidobacterium* in CKD specific diets reduced serum creatinine, urea nitrogen, and p-cresyl sulfate, demonstrating its role in reducing the accumulation of these uremic toxins [51]. Patients with advanced stages of CKD had increased numbers of *Eggerthella lenta* from the phyla *Actinobacteria*, *Fusobacterium nucleatum* from the phyla *Fusobacteriota*, and *Alistipes shahii* from the phyla *Bacteroidetes* [42].

CKD patients are predisposed to immune-mediated inflammatory disease [39,52]. An increase in *Actinomyces* and *Eggerthella* from the phyla *Actinobacteria*, *Clostridium III*, *Faecalicoccus*, and *Streptococcus* from the phyla *Firmicutes* coincides with inflammatory diseases, while *Gemmiger*, *Lachnospira*, and *Sporobacter* from the phyla *Firmicutes* are decimated [48]. In addition, in CKD the bacterial composition changes towards favouring proteolytic bacteria, which can produce protease enzyme and a reduction in the number of saccharolytic bacteria, which can break down sugars [53]. The major changes in gut microbial compositions and their pathophysiological functions under chronic kidney disease conditions are summarized in Table 2.

Diabetic kidney disease (DKD) is another complication often accompanying CKD. The gut microbiota modifies the endocrine functions of the gut and vice versa. Additionally, hyperglycemia moderates the alterations of the gut microbiota in DKD patients. The phyla *Proteobacteria*, *Verrucomicrobia*, and *Fusobacteria* are relatively more abundant in patients with DKD. Dysbiosis in both DKD and CKD patients results in similar downstream processes of uremic toxin accumulation-mediated progression of renal impairment [54]. *Akkermansia muciniphila* supplementation has various health benefits including aiding weight loss [55].

A systemic analysis of twenty-five studies shows that *Escherichia Shigella* and *Desulfovibrio* from the phylum *Proteobacteria* and *Streptococcus* from the phylum *Firmicutes* show an increased abundance in CKD patients. In addition, *Roseburia*, *Faecalibacterium* and *Prevotella_9* from the phyla *Firmicutes*, *Pyramidobacter* from the phyla Synergistota, and *Prevotellaceae_UCG-001* from the phyla *Bacteroidales* have a reduced abundance under CKD conditions [43].

**Figure 1:**
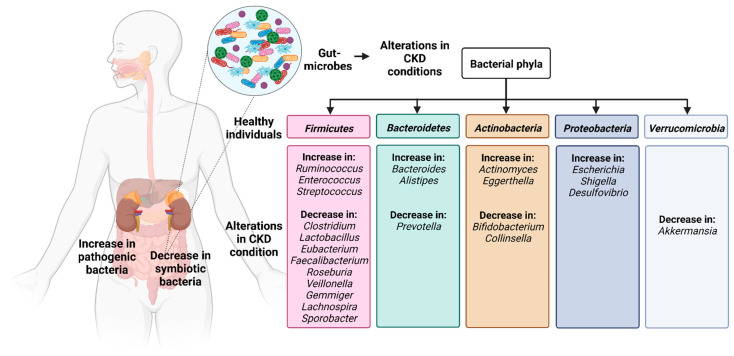
The human gut microbiome consists of five major phyla: *Firmicutes*, *Bacteroidetes*, *Actinobacteria*, *Proteobacteria*, and *Verrucomicrobia* within which there are alterations under CKD conditions. (Figure based on [3,4,39,40,41,42,43,44,46,48,53]).

Furthermore, antibiotics are administered to patients with CKD which alter the intestinal microflora [1]. The major CKD-induced changes in the gut microbiota are summarized in Figure 1.

## 3. Effects of Alterations in the Intestinal Flora in Chronic Kidney Disease

### 3.1. Production of Gut-Derived Metabolites

Alteration of intestinal microflora in CKD is a major source of alteration in the uremic metabolite profile [56,57,58]. Known uremic metabolites have been classified into three categories as proposed by the ´European Uremic Toxin Work Group´ (EUTox) based on their solubility and molecular weight: (a) small water-soluble molecules which are typically <500 Da, (b) protein-bound uremic toxins (PBUTs) which are typically more than 500 Da and (c) middle molecules which are ≥500 Da in size.

The two most investigated metabolites in CKD are p-cresyl sulfate and indoxyl sulfate. As the renal function declines, the concentration of these gut-derived uremic metabolites increases [56,57]. P-cresol/p-cresyl sulfate is produced by intestinal anaerobic bacteria due to the metabolism of phenylalanine and tyrosine [59]. Tryptophan is metabolized to indole by intestinal bacteria, which is then metabolized by the liver to indoxyl sulfate [60]. The concentrations of these two metabolites in the serum increase considerably (10-fold for p-cresyl sulfate and 50-fold for indoxyl sulfate) among patients with CKD [59]. This has severe negative impact on the health of the individual causing renal tubular cell damage, coagulation disturbances and endothelial dysfunction [61] and these two metabolites play a vital role in CKD progression [62]. Further, phenol and phenylacetic acid are produced by intestinal bacteria by degradation of tyrosine and phenylalanine, respectively [63]. Levels of polyamines such as spermine, and spermidine induce the maturation of small intestine mucosa and are cytotoxic in high quantities and are also elevated in CKD patients [63,64,65].

An increase in proteolytic bacteria and a reduction in saccharolytic bacteria lead to enhanced production of ammonia and uremic metabolites such as phenols and indoles, and a decrease in SCFAs [53]. This can be observed in CKD patients, where SCFAs concentration is decreased whereas p-cresyl sulfate and TMAO are increased [43].

Quaternary amines, such as choline/phosphatidylcholine, betaine, or l-carnitine, are metabolized by intestinal bacteria to produce TMAO [62]. In experimental animal models for CKD (C57BL6J mice) [66], a diet rich in choline or TMAO produces progressive tubulointerstitial fibrosis and renal dysfunction [67].

### 3.2. The Accumulation of Gut-Derived Metabolites

**Figure 2:**
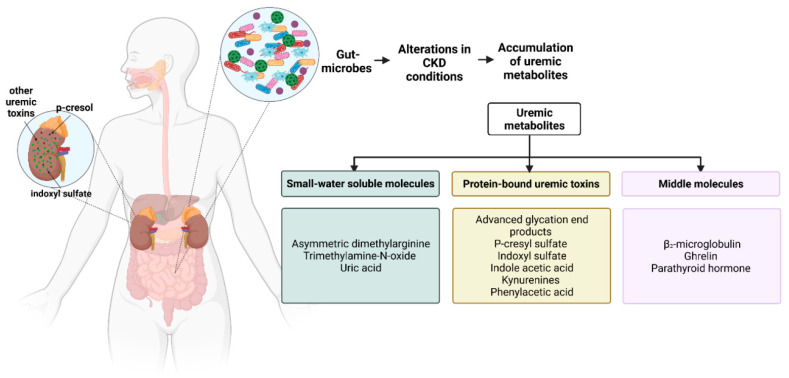
Known uremic metabolites have been classified into three categories based on their solubility and molecular weight: small water-soluble molecules, protein-bound uremic toxins (PBUTs) and middle molecules. Each category has characteristic representatives which have the highest global toxicity score in terms of the number of biological systems affected and overall experimental and clinical evidence [37].

Upon the production of the gut-derived uremic metabolites, a reduction in the filtration capacity of the kidneys increases the accumulation of these uremic, gut-derived metabolites in the serum, leading to toxicity, significantly increasing accelerated CKD progression and its accompanying co-morbidities such as atherosclerosis, obesity, and cardiovascular diseases [37]. The characteristic representatives of gut-derived metabolites are listed in Figure 2.

#### 3.2.1. Small Water-Soluble Molecules

Currently there are more than 30 known uremic metabolites which are classified as small water-soluble molecules. Characteristic representatives of this group include asymmetric dimethylarginine (ADMA), TMAO and uric acid which have the highest global toxicity score in terms of several biological systems affected and overall experimental and clinical evidence [37].

**Asymmetric dimethylarginine:** ADMA, an analogue of L-arginine, is produced by gut microbes and metabolized by dimethylarginine dimethylaminohydrolases-like enzymes in *Streptomyces coelicolor*, *Mycobacterium tuberculosis* from the phylum *Actinobacteria* and *Pseudomonas aeruginosa* from the phylum *Proteobacteria* among others [68]. ADMA downregulates nitric oxide (NO) production [69]: a vasoactive compound, which is essential for the maintenance of endothelial homeostasis. Low levels of NO are associated with impaired endothelial function impacting the lining of all major organs including the gut. Elevated ADMA in CKD patients leads to endothelial dysfunction and consequently cardiovascular events and is therefore identified as an independent risk factor for progression of atherosclerosis, cardiovascular death, and all-cause mortality [70].

**Trimethylamine-N-oxide:** TMAO is formed in the liver by oxidation of the gut microbe metabolite trimethylamine (TMA), which is produced by the fermentation of dietary nutrients such as choline, betaine or L-carnitine [71,72]. Even though the mechanism of TMAO production and its link to the gut microbiota is well established, the exact gut microbe species responsible are not known [73]; however, *Clostridium* XIVa strains, *Eubacterium* sp. strain AB3007 from the phylum *Firmicutes* and *Gammaproteobacteria* from the phylum *Proteobacteria* are likely candidates [74]. TMAO hinders mitochondrial function and energy metabolism by reducing the pyruvate and fatty acid oxidation and activates the release of IL-1β and IL-18 in endothelial cells, thereby promoting endothelial dysfunction which could in turn degrade the intestinal epithelial barrier [75]. Elevated TMAO production in CKD patients increases the risk of mortality by increasing endothelial dysfunction and cardiovascular events [72,75].

**Urea:** Urea is produced in the liver via the urea cycle, a catabolite of purine metabolism [76] from dietary amino acids and was traditionally considered to be biologically inert. Urea is then excreted to the gastrointestinal tract [77], where it is utilized by *E. coli* from the phylum *Proteobacteria* [78]. *Escherichia-Shigella* from the phylum *Proteobacteria* and *Bacteroides* from the phylum *Bacteroidetes* are opportunistic pathogens which increase in the gut with the increase in urea [79].

Urea has recently re-emerged as a highly relevant gut-derived toxin which triggers molecular changes leading to insulin resistance [80,81]. An increased concentration of urea leads to endothelial dysfunction by promoting free radical production, inhibiting glyceraldehyde-3-phosphate dehydrogenase (GAPDH) and upregulating protein kinase C isoform activity, which negatively impacts the gut endothelial lining. Endothelial pro-inflammatory pathways are initiated by urea through an increase in hexosamine pathway activity and inactivating an anti-atherosclerotic enzyme PGI2 synthase [82].

#### 3.2.2. Gut-Derived, Protein-Bound Uremic Toxins (PBUTs)

There are 25 known gut-derived PBUTs [83]. PBUTs are accumulated in CKD patients despite dialysis and hemofiltration due to their association with proteins which hinders their clearance from the plasma [84]. Characteristic representatives of this group include advanced glycation end products (AGEs), p-cresyl sulfate, indoxyl sulfate, indole acetic acid, kynurenines and phenylacetic acid which have the highest global toxicity score in terms of several biological systems affected and overall experimental and clinical evidence [37].

**Advanced glycation end products:** AGEs are characterized by non-enzymatically modified amino groups of proteins or lipids by monosaccharides. AGEs such as fructoselysine and N-ε-carboxymethyl lysine can be utilized by the gut microbes *E. coli* from the phylum *Proteobacteria*, *Intestinimonas* spp from the phylum *Firmicutes* and *Cloacibacillus* from the phylum *Synergistota* and potentially *Oscillibacter* spp from the phylum *Firmicutes*, respectively [85]. However, it is not known if these microbes are lower in CKD patients as compared to controls. AGEs cause sarcopenia and frailty in CKD patients [86]. The production and circulation of AGEs to tubular cells lead to high expression of transforming growth factor-beta (TGFβ), plasminogen activator inhibitor-1, tissue transglutaminase, and MCP1 contributing to tubulointerstitial fibrosis [87,88].

**p-Cresyl sulfate:** p-Cresyl sulfate is formed by the metabolism of aromatic amino acids by the gut microbiota. *Bacteroidaceae*, *Clostridiaceae*, *Enterobacteriaceae*, *Ruminococcaceae*, *Veillonellaceae*, *Lactobacillaceae*, *Staphylococcaceae*, *Lachnospiraceae* from the phylum *Proteobacteria*, *Bifidobacteriaceae* from the phylum *Actinobacteria*, *Enterococcaceae*, *Eubacteriaceae* from the phylum *Firmicutes*, *Porphyromonadaceae* from the phylum *Bacteroidetes* and *Fusobacteriaceae* from the phylum *Fusobacteriota* are the potential gut microbiota families which play a role in the production of p-cresyl sulfate [89].

p-Cresyl sulfate is less removed from circulation by dialysis as it is protein-bound, thereby significantly accumulating in serum of CKD patients [89]. In addition to causing cardiovascular complications p-cresyl sulfate is known to cause tubular cell damage, tubular epithelial–mesenchymal transition, tubulointerstitial inflammation and fibrosis, or whole-kidney damage by stimulating the renin–angiotensin–aldosterone system, TGFβ, intercellular adhesion molecule-1, ROS, and DNA methyltransferase 1 among other pathways [61].

**Indoxyl sulfate:** Indoxyl sulfate results from the metabolism of aromatic amino acids by numerous tryptophanase-producing bacteria in the gut, including *E. coli* from the phylum *Proteobacteria* [90], and *Lactobacillus* spp. from the phylum *Firmicutes* [91]. Indoxyl sulfate is not cleared from circulation by dialysis as more than 90% of this small molecule is bound to plasma proteins [92]. Indoxyl sulfate hinders glomerular filtration by promoting mesangial cell cytotoxicity by stimulating ROS production [93]. In addition, indoxyl sulfate is known to mediate renal fibrosis via the organic anion transporters/NADPH oxidase/ROS pathway through the mechanistic target of rapamycin complex 1 (mTORC1). mTORC1 mediates epithelial–mesenchymal transition of tubular epithelial cells (HK-2 cells), differentiation of fibroblasts into myofibroblasts (NRK-49F cells), and inflammatory response of macrophages [94].

**Indole acetic acid:** Indole acetic acid is a metabolite of aromatic amino acids metabolism [95], which is produced by numerous bacteria including *E.coli* from the phylum *Proteobacteria*, and is metabolized to Skatole by *Clostridium* from the phylum *Firmicutes*, *Bacteroides* from the phylum *Bacteroidetes* and to indole-3-aldehyde by *Lactobacillus acidophilus*, *Lactobacillus murinus*, *Lactobacillus reuteri* from the phylum *Firmicutes*, respectively [96,97,98]. In CKD patients, indole acetic acid positively correlates with inflammation and oxidative stress markers [99]. In addition to increasing ROS production, it activates the aryl hydrocarbon receptor (AhR)/p38MAPK/NF-κB pathway that induces proinflammatory enzymes [99].

**Kynurenines:** Kynurenine is a metabolite of tryptophan metabolism released via the kynurenine pathway. In silico analysis predicts the phylum *Actinobacteria*, *Firmicutes* and *Proteobacteria*, and genus *Bacteroides* from the phylum *Bacteroidetes*, *Fusobacteria* from the phylum *Fusobacteriota* as kynurenine-producing gut microbes [100].

Kynurenine promotes CKD pathophysiology through the inflammation-induced activity of indoleamine 2,3-dioxygenase [71] by regulating the AhR [101]. Proinflammatory factors such as interferon-gamma (IFN-γ) and TNF-α induce and transcriptionally enhance indoleamine dioxygenase [71] expression [102]. The accumulation of the kynurenine pathway metabolites leads to a depletion of tryptophan, which activates T regulatory cells inducing apoptosis of T cells inhibiting their proliferation and acting as a counter-reaction to inflammation [103].

**Phenylacetic acid:** Phenylacetic acid is formed from the microbial fermentation of aromatic amino acids, in addition to dietary intake [104]. *Bacteroides thetaiotaomicron*, *Bacteroides eggerthii*, *Bacteroides ovatus*, *Bacteroides fragilis*, *Parabacteroides distasonis* from the phylum *Bacteroidetes* and *Eubacterium hallii* and *Clostridium bartlettii* from the phylum *Firmicutes* found among the gut microbiota lead to significant phenylacetic acid production by fermentation [104].

Phenylacetic acid inhibits inducible nitric oxide synthase (iNOS) gene expression, reducing nitric oxide production, promoting cell proliferation and cytokine-induced endothelial expression of adhesion molecules and proinflammatory cytokines [105]. In addition, iNOS inhibition by phenylacetic acid reduces macrophage-killing aggravating immunodeficiency in CKD patients [106].

#### 3.2.3. Middle Molecules

There are 58 known middle molecules [107]. Characteristic representatives of this group include β₂-microglobulin, ghrelin and parathyroid hormone which have the highest global toxicity score in terms of a number of biological systems affected and overall experimental and clinical evidence [37]. However, ghrelin and the impact of microbiota on this uremic metabolite have not been studied in the pathogenesis of CKD conditions until now. A majority of the middle molecules are endogenously generated, as native proteins or in response to other gut-derived uremic metabolites [37]. These can be removed by dialysis but upon the use of high-flux membranes which can lead to conflicting results due to the loss of other unspecific metabolites [37].

**Β₂-microglobulin:** β₂-microglobulin is an essential endogenous protein produced by all nucleated cells. Animal studies show an increase in *Prevotella* spp. And *Bacteroides vulgatus* from the phylum *Bacteroidetes* and a decrease in *Rikenellaceae* also from the phylum *Bacteroidetes*, in transgenic animals expressing high β₂-microglobulin [108]. The 9 kDa from the β₂-microglobulin-derived metabolite, named shed β₂-microglobulin has antibacterial activity against *Staphylococcus aureus* from the phylum *Firmicutes*, leading to the production of ‘*Staphylococcus aureus*-shed β₂-microglobulin’ clumps which promote the migration of THP-1 monocytes [109]. Cell migration is associated with higher inflammation under CKD conditions [110]. Β₂-microglobulin causes dialysis-related amyloidosis in end-stage CKD, contributing to bone and joint deterioration in these patients [111]. The accumulation of β₂-microglobulin in the musculoskeletal system leads to amyloid fibrils formation instead of globular, roughly natively folded protein leading to loss of function in the musculoskeletal system [112].

**Parathyroid hormone:** Parathyroid hormone is a polypeptide that is synthesized and cleaved by the parathyroid gland into its active form which regulates calcium metabolism under normal physiological conditions [113]. Parathyroid hormone-dependent mineral metabolism requires butyrate production by intestinal microbiota [114]; however, the exact microbes involved and the mechanism by which this process is hindered in CKD patients have not been established so far [115]. In CKD patients, parathyroid hormone promotes serum fibroblast growth factor 23 (FGF23) and reduces vitamin D production and activity, resulting in CKD-mineral bone disorder [116]. The representative uremic toxins and the potential microbes which impact their concentrations in human gut are summarized in Table 3.

### 3.3. Deimmunization: Inflammation and Immunosuppression

Even though the human body contains numerous microbes they are kept outside the body by various inherent barriers such as the mucosal and epithelial layers. The overgrowth of pathogenic bacteria leads to increased secretion of endotoxins and other bacterial products such as lipopolysaccharides, peptidoglycans, outer membrane proteins and bacterial DNA, into the blood through the gut lining. This leads to alteration of intestinal permeability, the release of pro-inflammatory molecules and activation of the immune system associated with intestinal mucosa [38]. Along with an increase in pathogenic bacteria, there is enhanced production of inflammatory factors such as interleukin (IL)-6, IFN-γ and TNF-α, which would promote inflammation along the gut barrier [117]. This leads to chronic systemic inflammation, thereby elucidating the role of gut bacterial dysbiosis, leading to an increase in uremic toxins in circulation, promoting CKD progression [10]. Persistent systemic inflammation in CKD result from LPS-induced monocyte/macrophage activation and systemic inflammation by sepsis from Gram-negative bacterial species [52].

Contact with different members of the gut microbiota induces different types of immune responses in the host gut. Experimentally induced uremia in complementarity to bacterial DNA treatment leads to an increase in intestinal permeability, plasma hs-CRP, pentraxin-3, proinflammatory cytokines and IL-6 which are known biomarkers of inflammation [118,119]. While anaerobic *Clostridium* spp. from the phylum *Firmicutes*, usually found in the human gut, induces FoxP3+ regulatory T cells, an uncultivated segmented filamentous bacteria of the Clostridia family promotes T helper type 17 cell differentiation [120]. Lower levels of *Akkermansia* from the phylum *Verrucomicrobiota* in the gut of CKD patients coincide with higher levels of IL-10 and lower levels of *Lactobacillus* from the phylum *Firmicutes* are accompanied by higher levels of IL-4 and IL-10, demonstrating the impact of these gut microbes in modulating immune response in CKD patients [40]. The microbes involved in inflammation and immunosuppression and the possible mechanism are summarized in Table 4.

Peptidoglycan, a component of the cell wall of most Gram-negative and Gram-positive bacteria, translocates from the intestinal microflora triggering the nucleotide-binding oligomerization domain-containing protein 1 (NOD1) receptor which leads to activation of bone marrow neutrophils causing an immune response [121]. NOD1 is a soluble cytosolic receptor expressed in macrophages, vascular endothelial and smooth muscle cells. As pattern-recognition receptors, they recognize bacterial components such as peptidoglycan and lead to activation of pro-inflammatory components such as NF-ĸB [122]. Bacterial fermentation products such as SCFAs trigger G-protein-coupled receptors 41 and 43 on intestinal epithelial cells causing an enhanced production of cytokines and chemokines from these cells [123] and reducing the production of LPS-induced production of TNF-α, IL-1β and IL-6 [124].

**Figure 3:**
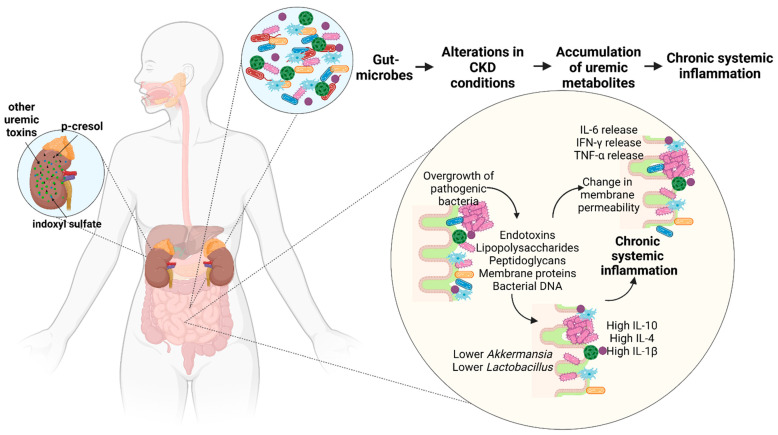
Gut microbes and their metabolites induce inflammation and mediate immunosuppression by interacting with various receptors leading to macrophage activation, dysregulation of chemokines and cytokines and promoting a change in membrane permeability. (Figure based on [1,38,40,52,117,123,124]).

Therefore, gut microbes and their products induce inflammation and mediate immunosuppression by interacting with various receptors on both endothelial cells and macrophages, which has been summarized in Figure 3.

### 3.4. Damage to Intestinal Epithelial Barrier

Impaired kidney function and reduced glomerular filtration rate in CKD patients lead to an increase in waste products of the kidney. Increased urea concentrations can be found in glandular secretions, e.g., saliva or gastric juice [1,125,126]. This leads to an influx of urea in the gastrointestinal tract, increasing hydrolyzation of urea to ammonia [CO(NH_2_)_2_ + H_2_O→CO_2_ + 2 NH_3_] and further conversion to ammonium hydroxide [NH_3_ +H_2_O→NH_4_OH]. Urea is hydrolyzed both spontaneously and by the microbial urease enzyme, resulting in an increase in gut pH, which promotes inflammation and eroding of the intestinal wall [125,126]. The urea-rich gut milieu further favors the growth of urease-containing microbial families. Twelve such families were identified in greater abundance in patients with end-stage renal disease. *Alteromonadaceae*, *Cellulomonadaceae*, *Clostridiaceae*, *Dermabacteraceae*, *Enterobacteriaceae*, *Halomonadaceae*, *Methylococcaceae*, *Micrococcaceae*, *Moraxellaceae*, *Polyangiaceae*, *Pseudomonadaceae* and *Xanthomonadaceae* [127]. Most bacterial urease occurs as inactive holoenzyme, consisting of a triple trimer with three active centers, each in one of the α-subunits. Nickel-ions act as a cofactor and are transported into the cells and integrated into the active site via UreD, UreE, UreF, UreG and UreH proteins [128].

Ammonia dampens the acid-dependent reinforcement of the epithelial barrier in gastric epithelial (he20) cells [129,130]. Ammonium hydroxide interacts with tight junction proteins facing the lumen and leads to the depletion of tight junction proteins occludin, claudin-1, and ZO-1 [131], lowering transepithelial electrical resistance, and increasing permeability of the intestinal wall. This metabolite further promotes the influx of pro-inflammatory leukocytes, promoting endocytosis of transcellular tight junction proteins [125,132]. The lowered concentration of butyrate-producing gut bacteria and consequently lowered levels of butyrate in CKD patients further destabilize the intestinal wall. Butyrate promotes the production of mucin and tight junction proteins [133,134,135] and possesses anti-inflammatory and antioxidant attributes; its absence thus promotes inflammation [134]. Macrophages in the intestine contribute to inflammation and bacterial translocation across the intestinal wall: LPS activate macrophages via TLR4. Activated macrophages produce the pro-inflammatory cytokines IL-6 and TNF-α, promoting microinflammation and contributing to the weakening of the integrity of the intestinal wall. One key function of macrophages is the phagocytosis of, for example, cellular debris or pathogens [136].

Phagocytosis is the process through which a cell engulfs an extracellular particle with its cell membrane and digests it. In conditions of disrupted intestinal barrier, macrophages pass through the barrier into the circulatory system, where they release waste products and possible endotoxins of phagocytized bacteria through exocytosis [137]. This would ultimately release endotoxins into the circulatory system, promoting endotoxemia and systemic inflammation [137]. The degradation of the epithelial tight junction complex weakens the transcellular connection, makes the epithelial cells more liable to mechanical stress, and has detrimental effects on cell polarity as well as the passive transcellular transport of ions and molecules, leading to an increase in luminal nutrients and water, promoting pathogen growth and dispersal [130,138].

Tight junction proteins from the apical junctional regions are dislocated by pathogens, resulting in their downward movement along the lateral membrane to intracellular locations. Alterations in tight junction fencing can lead to the flipping of basolateral proteins to apical sites, making new receptors available for microbes or toxins [130,138]. For example, *Escherichia coli* lines up along the junctions of epithelial cells and may use junctional proteins as receptors. The resulting alterations in pathogen-mediated signaling leads to actin perijunctional ring contraction, leading to enhanced paracellular permeability. Enhanced paracellular permeability provides an opportunity for the translocation of molecules by diffusion. In a parallel mechanism, cytoskeletal changes by microbes such as myosin light-chain phosphorylation trigger actomyosin contraction, also altering paracellular permeability [130,138].

In addition, fluid retention is a symptom of CKD which leads to adverse renal outcomes [139], with hemodialysis induced circulatory stress leading to increased endotoxin translocation from the gut [138]. The increased permeability of the intestinal wall results in the translocation of endotoxins and bacterial components such as DNA, LPS, and metabolites into the local and systemic tissue. This promotes activation of the pro-inflammatory cytokines IL-1 and IL-6 as well as expression of soluble TNF receptors which promote endothelial dysfunction, disturbing the endothelial barrier. The continuous activation of the immune system through the influx of bacterial components leads to an overactivation of the immune system and acquired immunodeficiency, as commonly seen in sepsis [140]. Products of bacterial catabolism in the circulatory system may also produce various uremic metabolites. The bacterial metabolites indole, trimethylamine, and p-cresyl glucoronidate are further metabolized in the liver to indoxyl sulfate, trimethylamine N-oxide, and p-cresyl glucuronide, respectively, contributing to kidney-related diseases [132].

**Figure 4:**
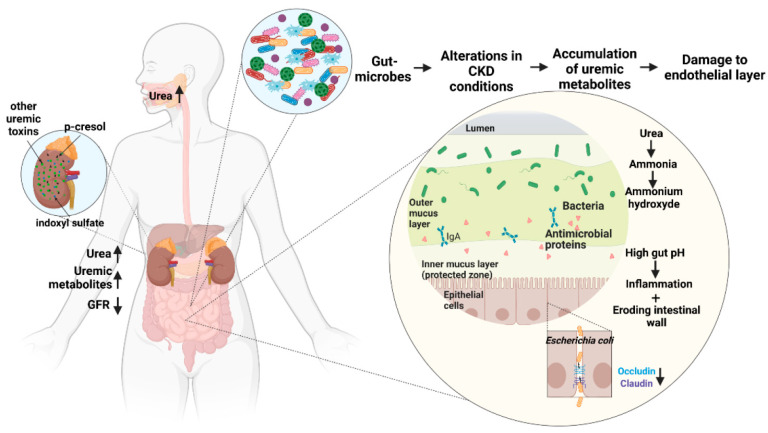
Gut microbiota-derived metabolites damage the intestinal epithelial barrier by an influx of urea in the gastrointestinal tract, increasing hydrolyzation of urea to ammonia and ammonium hydroxide, leading to depletion of tight junction proteins occludin and claudin-1, lowering transepithelial electrical resistance, and increasing permeability of the intestinal wall. (Figure based on [1,125,126,130,132,135]).

In addition, CKD patients undergoing extracorporeal treatment show increased levels of circulating endotoxins compared to CKD patients in a similar stage of renal failure but without extracorporeal treatment. Endotoxemia and chronic inflammation through intestinal barrier dysfunction are promoted by hypotension, which is a concomitant of hemodialysis [141] and promote bowel edema and ischemia [142]. Extracorporeal treatment-induced ischemia and hypoxia are additional risk factors since the intestinal wall is sensitive to low oxygen milieu due to epithelial injuries resulting from hypoxic villus tips [143,144]. Hemodialysis furthermore leads to a rise in core temperature, which could disturb the intestinal barrier. Diuretics and anticoagulants used in hemodialysis are potential risk factors, promoting hypotension and micro-bleeding, respectively [125,138,144]. The mechanisms by which the gut microbiota damages the intestinal epithelial barrier have been summarized in Figure 4.

These results emphasize the influence of CKD on the intestinal barrier and how dysfunction of the latter can in turn worsen the effect of CKD, creating a feedforward loop.

## 4. Discussion

The human gut microbiome consists of bacterial species from five major bacterial phyla, *Firmicutes*, *Bacteroidetes*, *Actinobacteria*, *Proteobacteria* and *Verrucomicrobia*. The ratio of *Firmicutes/Bacteroidetes*, which are the most abundant phyla in the human gut, plays an important role in maintaining intestinal homeostasis. Some species of *Actinobacteria* have been used as probiotics. An increase in *Verrucomicrobia* coincides with a healthy gut, while an increase in *Proteobacteria* coincides with dysbiosis and pathogenic conditions.

In CKD patients among members of the phylum *Firmicutes*, there is an increase in *Ruminococcus*, *Enterococcus* and *Streptococcus* and a decrease in *Clostridium*, *Lactobacillus*, *Eubacterium*, *Faecalibacterium*, *Roseburia*, *Veillonella*, *Gemmiger*, *Lachnospira* and *Sporobacter.* Among members of the phylum *Bacteroidetes*, there is an increase in *Bacteroides* and *Alistipes* and a decrease in *Prevotella*. Among members of the phylum *Actinobacteria* there is an increase in *Actinomyces* and *Eggerthella*, along with a decrease in *Bifidobacterium* and *Collinsella*. There is an increase in the members of the phylum *Proteobacteria*, especially genus *Escherichia*, *Shigella* and *Desulfovibrio*. From the phylum *Verrucomicrobia* the genus *Akkermansia* is decreased in CKD patients.

In addition, CKD-associated alteration of this intestinal microbiome results in metabolic changes and the accumulation of uremic metabolites, which have a feedforward adverse effect on CKD patients, inhibiting renal functions and increasing comorbidities such as atherosclerosis and CVD. Characteristic representatives of small water-soluble uremic toxins include ADMA, TMAO and urea which are most influenced by members of the phyla *Firmicutes*, *Actinobacteria* and *Bacteriodetes.* Characteristic representatives of protein-bound uremic toxins include AGEs, p-cresyl sulfate, indoxyl sulfate, indole acetic acid, the kynurenines and phenylacetic acid which are most influenced by members of the phyla *Firmicutes*, *Proteobacteria* and *Bacteriodetes.* Characteristic representatives of middle molecules include β₂-microglobulin, ghrelin and parathyroid hormone which are most influenced by members of the phyla *Firmicutes* and *Bacteriodetes*. Elevated levels of endotoxins in the gut lead to endotoxemia and inflammation, further accelerating CKD progression. Gut microbes and their metabolites induce inflammation and mediate immunosuppression by interacting with various receptors leading to macrophage activation, dysregulation of chemokines and cytokines and promoting a change in membrane permeability. Gut microbiota-derived metabolites damage the intestinal epithelial barrier by an influx of urea in the gastrointestinal tract, increasing hydrolyzation of urea to ammonia and ammonium hydroxide, leading to depletion of tight junction proteins occludin and claudin-1, lowering transepithelial electrical resistance, and increasing permeability of the intestinal wall.

Even though the gut microbiota and their importance in the progression of CKD have gained traction in recent years, there is still a knowledge gap that hinders widespread therapeutic application. Most clinical studies focus on the links between CKD, uremic metabolites and the microbes dysregulated without focusing on the mechanisms by which these microbes regulate CKD progression. Moreover, the studies linking microbes to uremic metabolite concentrations are limited by lack of standardized techniques for quantifying the large array of diverse uremic metabolites. Comprehensive systematic studies are required to completely understand the role of the gut microbiota in CKD progression, followed by gut microbiota-corrective studies to restore healthy gut conditions. In addition, pathogen-induced deimmunization and intestinal barrier disruption present novel opportunities for mitigating CKD progression.

## 5. Conclusions

Alterations in the members of these phyla alter the total gut microbiota, with a decline in the number of symbiotic flora and an increase in the pathogenic bacteria, causing or aggravating CKD. Numerous studies focus on the alterations of the gut microbiota in CKD and the alteration of metabolic profile of CKD patients. However, there is a lack of studies connecting these two modifications in high-throughput studies. Gut-derived uremic metabolites exhibit cell-damaging properties, damage the intestinal epithelial cell wall, increase gut permeability and lead to the translocation of bacteria and endotoxins from the gut into the circulatory system. To limit CKD progression, it is imperative to address the changes in the gut microbiome; therefore, it is necessary to identify and characterize the role of different microbes in the progression of CKD.

## Figures and Tables

**Table 1:** Functions of gut microbes from the five most abundant phyla in the human gut.

Phyla	Prominent Members	Functions
*Firmicutes*	*Ruminococcus*, *Clostridium*, *Lactobacillus*, *Anaerostipes*, *Eubacterium*, *Faecalibacterium*, and *Roseburia*	- Break down complex carbohydrates in the gut that cannot be digested by endogenous enzymes [7] - SCFA production [8] - Production of antimicrobial, anti-carcinogenic and anti-inflammatory molecules and peptides [7] - Increase in firmicutes coincides with obesity [14]
*Bacteroidetes*	*Bacteroides*, *Prevotella*, *Clostridiales* and *Xylanibacter*	- Ferment complex carbohydrates and produce volatile fatty acids which are a source of energy for the host [9] - Promote the growth of mutualistic bacteria upon high-fiber consumption [11] - Metabolic changes in the microbiota, leading to reduced IL 18 production, mucosal inflammation, and potential systemic autoimmunity [12] - An increase in *Bacteroidetes* coincides with inflammatory bowel disease [14]
*Actinobacteria*	*Bifidobacteria*	- Essential for gut homeostasis - Probiotic [20]
*Proteobacteria*	*Escherichia coli* and *Salmonella*	- A dysbiotic increase leads to a compromised gut microbiota and inflammation [21]
*Verrucomicrobia*	*Akkermansia muciniphila*	- Improves gut barrier function and has anti-inflammatory properties [22]

**Table 2:** Major changes in gut microbial compositions and their pathophysiological functions under chronic kidney disease conditions.

Phyla	Changes in Microbes	Changes in Functions
*Firmicutes*	Lower *Lactobacillus*, *Roseburia*, *Faecalibacterium*, *Prevotella*, *Gemmiger*, *Lachnospira*, and *Sporobacter* Increase in *Streptococcus*, *Clostridium III*, *Faecalicoccus*	- Lower *Lactobacillus* is associated with hypertension and linked to adverse outcomes in patients with CKD [42]. - Less production of butyrate a compound known to protect kidneys [44]. - *Ruminococcus* promotes inflammatory bowel syndrome, produces inflammatory polysaccharides such as glucorhamnan [47]. - Higher protease production, lower saccharolysis [53]. - Leading to damaged local gastrointestinal tract function and aggravating inflammation [49].
*Bacteroidetes*	Minor differences in *Paraprevotella Alloprevotella*	- Lower levels of *Bacteroidetes* are associated with lower SCFA production [16].
*Actinobacteria*	Decrease in *Bifidobacteria* Increase in *Eggerthella lenta* and *Actinomyces*	- Supplementing *Bifidobacterium* reduced serum creatinine, urea nitrogen, and p-cresyl sulfate [51].
*Proteobacteria*	Increase in *Escherichia*, *Shigella*, *Desulfovibrio*	- Inflammatory response, alteration of gut mucosal permeability and increasing the cell ratio of intestinal T helper 17 cells to T regulatory cells and promoting the LPS translocation [41].
*Verrucomicrobia*	Decrease in *Akkermansia muciniphila*	- Proportionate reduction in functions such as gut-barrier function and thickness of the mucus [4] and the detoxification of hydrogen sulfide [39], the growth of bacteria-producing SCFAs and energy produced as a result of mucus degradation [40].

**Table 3:** Characteristic representatives, of the three uremic toxin classes, based on their global toxicity score in terms of the number of biological systems affected and overall experimental and clinical evidence [37] and the microbes which influence their abundance in the human gut.

Uremic Metabolite Class	Representative Molecules	Potential Microbes Involved
Small water-soluble molecules	Asymmetric dimethylarginine	*Streptomyces coelicolor*, *Mycobacterium tuberculosis* from the phylum *Actinobacteria* and *Pseudomonas aeruginosa* from the phylum *Proteobacteria* among others [65]
	Trimethylamine-N-oxide	*Clostridium* XIVa strains, *Eubacterium* sp. strain AB3007 from the phylum *Firmicutes* and *Gammaproteobacteria* from the phylum *Proteobacteria* are likely candidates [67]
	Urea	Utilized by *E. coli* from the phylum *Proteobacteria* [71]. *Escherichia-Shigella* from the phylum *Proteobacteria* and *Bacteroides* from the phylum *Bacteroidetes* increase with the increase in urea [72].
Protein-bound uremic toxins	Advanced glycation end products	Utilized by the gut microbes *E. coli* from the phylum *Proteobacteria*, *Intestinimonas* spp from the phylum *Firmicutes* and *Cloacibacillus* from the phylum *Synergistota* and potentially *Oscillibacter* spp from the phylum *Firmicutes* [78]
	P-cresyl sulfate	*Bacteroidaceae*, *Clostridiaceae*, *Enterobacteriaceae*, *Ruminococcaceae*, *Veillonellaceae*, *Lactobacillaceae*, *Staphylococcaceae*, *Lachnospiraceae* from the phylum *Proteobacteria*, *Bifidobacteriaceae* from the phylum *Actinobacteria*, *Enterococcaceae*, *Eubacteriaceae* from the phylum *Firmicutes*, *Porphyromonadaceae* from the phylum *Bacteroidetes* and *Fusobacteriaceae* from the phylum *Fusobacteriota* play a role in the production of p-cresyl sulfate [82]
	Indoxyl sulfate	Produced by *E. coli* from the phylum *Proteobacteria* [87], and *Lactobacillus* spp. from the phylum *Firmicutes* [88]
	indole acetic acid	Produced by numerous bacteria including *E.coli* from the phylum *Proteobacteria*, and is metabolized to Skatole by *Clostridium* from the phylum *Firmicutes*, *Bacteroides* from the phylum *Bacteroidetes* and to indole-3-aldehyde by *Lactobacillus acidophilus*, *Lactobacillus murinus*, *Lactobacillus reuteri* from the phylum *Firmicutes*, respectively [89,90,91]
	Kynurenines	*In silico* analysis predicts the phylum *Actinobacteria*, *Firmicutes* and *Proteobacteria*, and genus *Bacteroides* from the phylum *Bacteroidetes*, *Fusobacteria* from the phylum *Fusobacteriota* as kynurenine-producing gut microbes [97]
	Phenylacetic acid	*Bacteroides thetaiotaomicron*, *Bacteroides eggerthii*, *Bacteroides ovatus*, *Bacteroides fragilis*, *Parabacteroides distasonis* from the phylum *Bacteroidetes* and *Eubacterium hallii*, *Clostridium bartlettii* from the phylum *Firmicutes* lead to higher phenylacetic acid production [101]
Middle molecules	β₂-microglobulin	An increase in *Prevotella* spp. And *Bacteroides vulgatus* from the phylum *Bacteroidetes* and a decrease in *Rikenellaceae* also from the phylum *Bacteroidetes* [101]
	Parathyroid hormone	The exact microbes involved have not been established so far [112]

**Table 4:** The microbes involved in inflammation and immunosuppression.

Microbes	Role in Inflammation and Immunosuppression
*Clostridium* spp. from the phylum *Firmicutes*	Induces FoxP3+ regulatory T cells [120]
Uncultivated segmented filamentous bacteria of the Clostridia family	Promotes T helper type 17 cell differentiation [120]
*Akkermansia* from the phylum *Verrucomicrobiota*	Higher levels of IL-10 [40]
Lower levels of *Lactobacillus* from the phylum *Firmicutes*	Higher levels of IL-4 and IL-10 [40]

## Data Availability

Not applicable.
